# The Impact of Down-Regulation on Obstetrics and Perinatal Outcomes in Singleton Pregnancies After *In Vitro* Fertilization

**DOI:** 10.3389/fendo.2021.622081

**Published:** 2021-03-10

**Authors:** Lei Jin, Jihui Ai, Yu Zheng, Biao Chen, Lan Wang, Xiyuan Dong

**Affiliations:** Reproductive Medicine Center, Tongji Hospital, Tongji Medical College, Huazhong University of Science and Technology, Wuhan, China

**Keywords:** down-regulation, obstetrics outcomes, perinatal outcomes, singleton pregnancy, *in vitro* fertilization

## Abstract

**Background:**

down-regulation has been widely used in IVF treatment; however, it lacks reports on the impact of down-regulation on obstetrics and perinatal outcomes. The purpose of this study was to compare the obstetrics and perinatal outcomes among different down-regulation conditions.

**Methods:**

this is a retrospective cohort study on 3578 patients achieving cumulative singleton clinical pregnancy after their first oocytes retrieval cycle. Patients were grouped according to the serum estradiol after down-regulation (E2D) into three groups: <30, 30-55, >55 pg/ml. The obstetrics and perinatal outcomes, and live-birth rate per clinical pregnancy were main outcome measures. In the subgroup analysis, patients were further divided according to the mode of transfer. ANOVA, chi-square test, multivariate logistic regression, and multivariate general linear model were performed for statistical analysis.

**Results:**

the patients with E2D <30, 30-55, >55 pg/ml had similar live-birth rates. The patients with E2D <30 pg/ml had a lower risk of hypertension disorders than those with E2D 30-55 pg/ml. No difference was found in the risks of placenta previa, placenta abruption, premature rupture of membrane, hemorrhage, gestational diabetes mellitus, or intrauterine growth restriction. The newborns in the group with E2D <30 pg/ml had a lower risk of PICU admission than those in the group with E2D >55 pg/ml. There was no difference in the risks of congenital anomalies or mortality among the three groups. No differences were found in the gestational week, percentages of preterm birth and very preterm birth, birth weight, percentages of low birth weight and very low birth weight, delivery mode, or sex of newborn. Subgroup analysis showed that E2D 30-55 pg/ml was associated with a higher risk of low birth weight in patients with one fresh transfer + frozen transfer(s).

**Conclusion:**

Down-regulation has no effect on the live-birth rate per clinical pregnancy. Patients with E2D <30 pg/ml may have advantages regarding lower risks of both maternal hypertension and newborn PICU admission. E2D 30-55 pg/ml may be associated with low birth weight in patients with relatively low quality embryos.

## Introduction

Pituitary down-regulation with a gonadotropin-releasing hormone-agonist (GnRH-a) is common practice in the field of *in vitro* fertilization (IVF). Down-regulation can avoid a premature luteinizing hormone (LH) surge, favor follicle development, synchronize the growth of the follicles and endometrium, and thus improve IVF success (1). In addition, down-regulation has an advantage regarding treatment scheduling. Previous studies have shown the superiority of down-regulation, in terms of a lower cycle cancelation rate and a higher pregnancy rate (2). Our previous study showed that the degree of down-regulation was associated with ovarian response, clinical pregnancy rate, and live birth rate. A serum estradiol (E2) level after down-regulation (E2D) <55 pg/ml represents the optimal status for subsequent pregnancy (3).

It is estimated that births after IVF account for over 1% of all births in the UK (4). There is a growing concern about the safety of IVF, in terms of both obstetrics and perinatal outcomes (5–7). It has been reported that IVF increases the risks of maternal disorders such as placenta accreta, hypertensive disorders, and psychological disorders (5, 8, 9). Besides, IVF has been reported to be associated with preterm birth (PTB), low birth weight (LBW), gender bias (10–12), and congenital anomalies (13).

To our knowledge, there is a lack of studies analyzing IVF safety in different down-regulation conditions. The objective of this study was to evaluate whether there are associations between down-regulation and obstetrics/perinatal outcomes in singleton pregnancies after IVF.

## Materials and Methods

### Study Population

This is a retrospective cohort study on patients who underwent their first IVF treatment at our center, between January 2009 and December 2013. Inclusion criteria: (1) patients undergoing standard mid-luteal phase GnRH-a long protocol and (2) patients who achieved clinical singleton pregnancy after their first stimulated cycle, including fresh and/or frozen embryo transfer (FET). Exclusion criteria: (1) patients who underwent IVF involving donation or freezing of gametes, (2) patients who underwent preimplantation genetic testing (PGT), and (3) patients with hypertension, diabetes mellitus, or immune disorders. All patients were followed up to the end of pregnancy. In total, the data on 3,578 patients were extracted for analysis. There were three modes of transfer among the patients in this study: (1) one fresh transfer only, (2) freezing all + subsequent frozen transfer(s), and (3) one fresh transfer + subsequent frozen transfer(s). In subgroup analyses, the patients were analyzed according to the mode of transfer. This study was conducted with the formal approval of the Institutional Review Board (IRB) of Tongji Hospital. All patients in this study gave written consent regarding the inclusion of data pertaining to them. The data were fully anonymized before analysis.

### Clinical Protocols

Down-regulation, ovarian stimulation, IVF, embryo culturing, and embryo transfer were performed as previously described (3). Briefly, a daily injection of 0.1 mg GnRH-a (Decapeptyl, Ferring, Switzerland, or Diphereline, Ipsen, Australia) was initiated in the mid-luteal phase of the preceding cycle. Ovarian stimulation with gonadotropin was initiated with recombinant follicle-stimulating hormone (rFSH; Gonal-F, Serono, Switzerland, or Puregon, Organon, Netherlands). The starting dose was 150–225 IU/d based on age, antral follicle count (AFC), basal FSH, and body mass index (BMI). The dosage of GnRH-a was then reduced to 0.05 mg/d until the day of human chorionic gonadotropin (hCG) trigger. The gonadotropin dose was adjusted according to the ovarian response, which was assessed based on serum E2, progesterone (P), LH, and serial ultrasound scans. When at least 2–3 follicles developed to a diameter ≥18 mm, 10,000 IU hCG was given to trigger the maturation of follicles. Oocytes were retrieved transvaginally at 36–38 h after the hCG injection. The fertilization method involved IVF and intracytoplasmic sperm injection (ICSI). The morphology of each of the day 3 embryos was scored as top, good, fair or poor, according to the British Fertility Society (BFS) and Association of Clinical Embryologists (ACE) cleavage-stage embryo grading system (14). In a fresh cycle, no more than two best-quality embryos were transferred on day 3 after oocyte retrieval. There were three levels of embryo transferred: transfer with ≥1 top embryo(s), transfer with ≥1 good embryo(s), and transfer with ≥1 fair embryo(s). Poor embryos were not used for transfer. Luteal phase support was provided from the day of oocyte retrieval until the 10^th^ week of gestation, with 60 mg/d intramuscular P (P injection, Xianju, China) or 90 mg/d vaginal P (8% Crinone, Merck, UK).

### Blastocyst Culture

Excess cleavage-stage embryos were cryopreserved, or cultured to the blastocyst stage and then cryopreserved for subsequent FET. The decision regarding whether to perform extended culture or not was made by the doctor and patient together. If a patient was concerned about having no or very few available embryos after extended culture, typically two day 3 embryos were frozen, while the other embryos were further cultured. G2 medium (Vitrolife, Sweden) was used for blastocyst culture. CO_2_, O_2_, and N_2_ were maintained at 6, 5, and 89% in an incubator (CO_2_ incubator C60, Labotect, Germany, or K-MINC-1000, Cook, Australia). The embryos were further cultured at 37°C until day 5 or 6. The blastocysts were scored using Gardner and Schoolcraft’s grading system (15).

### Embryo Vitrification and Warming

Embryo vitrification and warming were performed as previously described (16). The embryos were vitrified within 2 h after scoring. The entire vitrification procedure was performed at room temperature (22–25°C). The embryos were equilibrated in equilibration solution (ES; Vitrification kit, Kitazato, Japan), containing 7.5% ethylene glycol and 7.5% dimethylsulfoxide (DMSO), for 5–10 min. The embryos were then transferred into vitrification solution (VS; Vitrification kit, Kitazato, Japan), which contained 15% ethylene glycol, 15% DMSO, and 0.5 mol/L sucrose, and they were subsequently loaded onto the surface of a Cryotop System (Kitazato, Japan) within 40–60 s. They were then immediately submerged in liquid nitrogen.

On the day of transfer, embryos were warmed at room temperature (22–25°C). They were transferred to thawing solution (TS; Vitrification kit, Kitazato, Japan), which contained 1.0 mol/L sucrose, for 1 min, followed by 3 min in diluent solution (DS; Vitrification kit, Kitazato, Japan), which contained 0.5 mol/L sucrose. They were then washed twice in washing solution 1 and 2 (WS1 and WS2; Vitrification kit, Kitazato, Japan) for 5 min each. The warmed embryos were then cultured for at least 2 h before post-warming evaluation. The temperature of the TS, WS2, and culture media were maintained at 37°C. After warming, the embryos were checked for survival under an inverted microscope. They were immediately transferred after post-warming evaluation.

### Endometrial Preparation and Frozen Embryo Transfer

The endometrial preparation in a FET cycle was performed using a natural cycle (NC) or an artificial cycle (AC) protocol. Regarding the NC protocol, serial trans-vaginal ultrasound scans were performed until the endometrial thickness reached ≥8 mm or approximated the level in the stimulated cycle. The timing of ovulation was estimated by a combined analysis of ultrasound results, the LH level and the P level. Regarding the AC protocol, E2 valerate tablets (PROGYNOVA, Bayer, Germany) were administered at 2 mg/d on day 1–4, 4 mg/d on day 5–8, and 6 mg/d on day 9–12. Serial ultrasound scans were performed from day 10–12. The dosage was adjusted based on the endometrial thickness. When the endometrial thickness reached ≥8 mm or approximated the level in the stimulated cycle, 60 mg P was used to transform the endometrium. FET was performed 3 or 5 days after the transformation, according to the day of embryo development. In both the NC and AC protocols, luteal phase support was provided from the day of transfer until the 10^th^ week of gestation, with 60 mg/d intramuscular P (P injection, Xianju, China) or 90 mg/d vaginal P (8% Crinone, Merck, UK).

### Outcome Measures

The primary outcome measures were maternal and neonatal outcomes. Maternal complications included hypertension (International Classification of Diseases (ICD) 10 codes O13-15), gestational diabetes mellitus (O24), intrauterine fetal restriction (O36), premature abruption of membrane (O42), placenta previa (O44), placenta abruption (O45), and postpartum hemorrhage (O72). Neonatal outcomes included gestational week, PTB (<37 weeks of gestation), very preterm birth (VPTB, <32 weeks of gestation), birth weight, LBW (< 2,500 g), very low birthweight (VLBW, < 1,500 g), delivery mode, sex of newborn, congenital anomalies, pediatric intensive care unit (PICU) admission, and mortality. The ovarian stimulation performance and live-birth rate per clinical pregnancy were also analyzed. A clinical pregnancy was diagnosed when the serum hCG level reached >20 IU/l at 2 weeks after transfer and the gestational sac was detected on ultrasound at 5–7 weeks after transfer. A live-birth was defined as complete expulsion or extraction of a live baby after the 28^th^ week of gestation (17).

### Grouping of Patients With Estradiol After Down-Regulation

Patients were classified into the profound (E2D <30 pg/ml), medium (E2D 30–55 pg/ml), and insufficient (E2D >55 pg/ml) down-regulation groups, according to the criteria published in our previous study. Serum LH after down-regulation (LHD) was not used for patient grouping, because this study showed no significant effect of LHD on the cumulative clinical pregnancy rate or cumulative live birth rate per retrieval cycle (3).

### Statistical Analysis

SAS 9.2 (SAS Inc., NC, USA) and STATA 14 (STATA Inc., TX, USA) were used for statistical analysis. Continuous variables are presented as mean ± SD. Categorical variables are presented as number (percentage). Analysis of variance (ANOVA) and the chi-square test were performed, as appropriate. Multiple comparisons were performed using Tukey’s test and the Bonferroni correction. Multivariate logistic regression was used to analyze the effect of E2D on categorical outcomes, including live-birth, PTB, LBW, delivery mode, and sex of newborn, with age, BMI, infertility type, infertility cause, number of oocytes retrieved, mode of transfer, day 3 versus day 5 embryo, endometrial thickness at transfer, and LHD as covariates. Multivariate general linear models were used to evaluate the associations between E2D and continuous outcomes, including gestational week and birthweight, with the same covariates as in the logistic regression models. Multivariate analysis was not applicable for analyzing the obstetrics complications, VPTB, VLBW, congenital anomalies, PICU admission, or mortality, because of the low incidences of these conditions. A *P* value < 0.05 was considered statistically significant.

## Results

**Figure 1:**
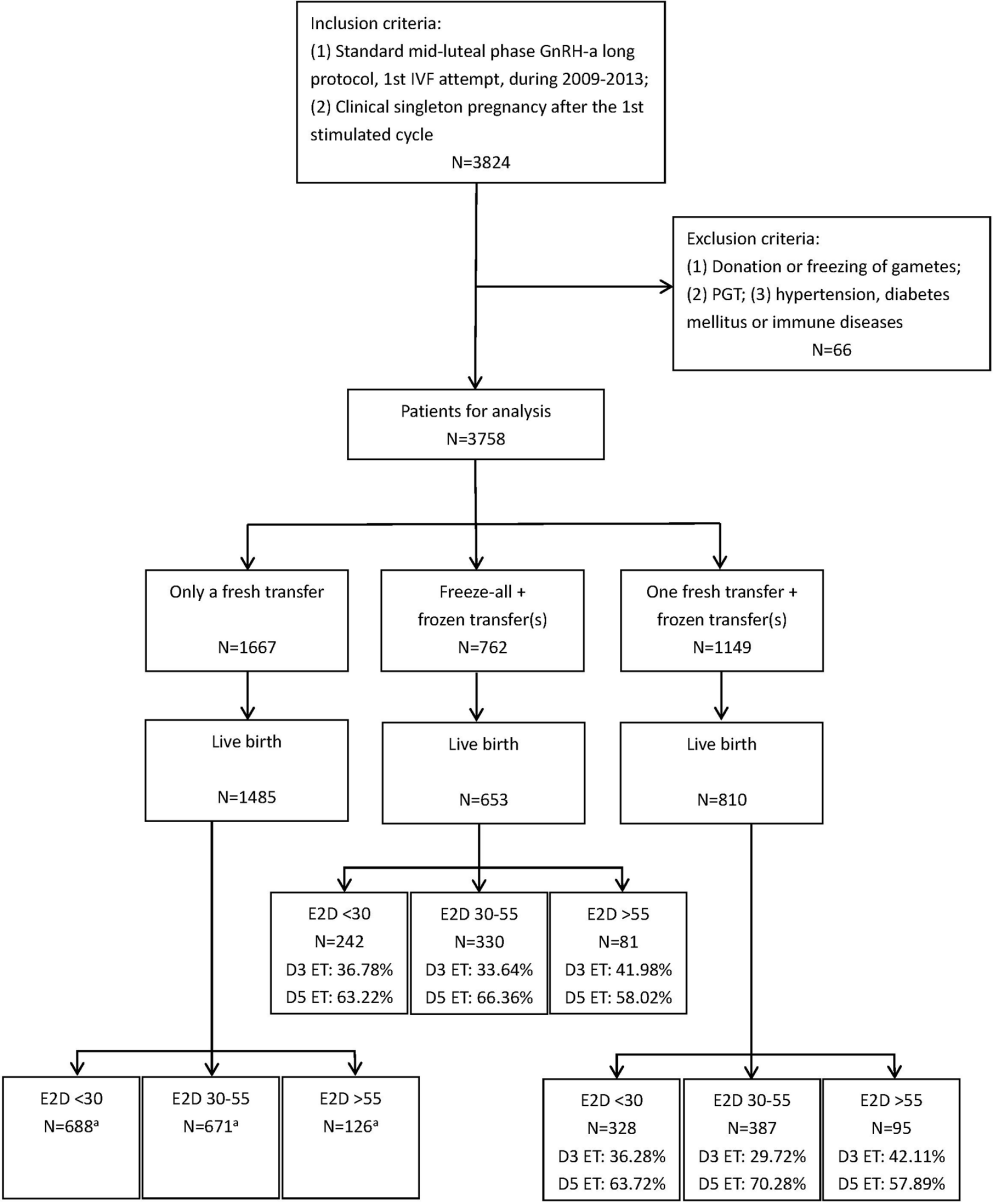
The flow chart of patient selection. ^a^All embryos transferred in these subgroups were day 3 cleavage-stage embryos.

**Figure 1** shows the patient selection process in this study. Data from 3,578 patients were used in the final analysis. The demographic and clinical characteristics are shown in **Table 1**. There were significant differences in the age, BMI, baseline serum FSH level, and AFC among the patients with E2D <30, 30–55, and >55 pg/ml. The duration and type of infertility, and infertility causes, were similar among the three groups.

**Table 1:** The demographic and clinical characteristics.

	E2D <30 pg/ml	E2D 30–55 pg/ml	E2D >55 pg/ml	*P* value
No. of patients	1,523	1,677	378	
Age (years)	30.41 ± 4.12	29.94 ± 4.00	30.16 ± 3.99	<0.01
BMI (kg/m^2^)	21.24 ± 2.61	21.58 ± 2.93	22.22 ± 3.12	<0.01^abc^
Duration of infertility (years)	4.57 ± 3.24	4.46 ± 3.18	4.85 ± 3.39	NS
Type of infertility, n (%)				NS
Primary infertility	749 (49.2)	768 (45.8)	178 (47.1)	
Secondary infertility	774 (50.8)	909 (54.2)	200 (52.9)	
Infertility cause, n (%)				NS
Tubal factors	762 (50.05)	809 (48.24)	167 (44.18)	
Endometriosis	90 (5.91)	121 (7.22)	21 (5.56)	
Male factors	245 (16.09)	281 (16.76)	61 (16.14)	
Oligo- or anovulation	251 (16.48)	300 (17.89)	87 (23.02)	
Unexplained factors	97 (6.37)	89 (5.31)	22 (5.82)	
Diminished ovarian reserve	24 (1.58)	30 (1.79)	7 (1.85)	
Other factors	54 (3.55)	47 (2.80)	13 (3.44)	
Basal FSH level (mIU/ml)	6.42 ± 1.92	6.11 ± 1.86	5.67 ± 1.83	<0.01^abc^
Basal AFC	14.31 ± 5.35	15.30 ± 5.85	16.78 ± 6.94	<0.01^abc^

E2D, estradiol after down-regulation; BMI, body mass index; FSH, follicle stimulating hormone; AFC, antral follicle count; NS, not significant; ^a^E2D <30 pg/ml vs. 30–55 pg/ml; ^b^E2D <30 pg/ml vs. > 55pg/ml; ^c^E2D 30–55 pg/ml vs. >55 pg/ml.

**Supplementary Figure 1** shows the results of multivariate logistic regression of live-birth, with E2D as a continuous variable. E2D exhibited no significant association with the probability of live-birth. However, restricted cubic spline analysis showed a trend of decreased probability of live-birth with increasing E2D. In addition, there were two potential E2D cut-off points: 30 and 55 pg/ml.

**Table 2** shows the ovarian stimulation parameters and pregnancy outcomes. There were pairwise significant differences in LHD and mode of transfer among the three E2D groups. Additionally, the patients with E2D <30 pg/ml had a higher stimulation dosage compared to those with E2D >55 pg/ml (29.00 ± 6.79 vs. 25.92 ± 7.91 ampules, *P* < 0.01). The serum peak E2 level was lower in the patients with E2D <30 pg/ml compared to those with E2D 30–55 or >55 pg/ml (4,982.93 ± 2,792.94 vs. 5,471.74 ± 2,890.84 and 5,623.62 ± 2,614.19 pg/ml, *P* < 0.01). Significant differences were also found in the duration of rFSH, number of oocytes retrieved, and number of available embryos. The serum P level and endometrial thickness at transfer were similar among the three E2D groups. The patients in the three E2D groups had similar live-birth rates. The patients were further sub-divided into three subgroups according to the mode of transfer, and E2D had no significant effect on live-birth in any of the subgroups.

**Table 2:** The ovarian performance and pregnancy outcomes.

	E2D <30 pg/ml	E2D 30–55 pg/ml	E2D >55 pg/ml	*P* value
No. of patients	1,523	1,677	378	
E2D (pg/ml), (mean ± SD) (median and range)	19.61 ± 6.81 20.60 (0.07–29.97)	40.63 ± 6.91 40.00 (30.00–54.99)	65.12 ± 10.16 62.0 (55.00–114.10)	<0.01^cde^
LHD (mIU/ml), (mean ± SD) (median and range)	1.55 ± 1.06 1.31 (0.07–13.00)	1.83 ± 1.26 1.51 (0.05–13.90)	2.51 ± 1.73 1.92 (0.38–13.66)	<0.01^cde^
Duration of rFSH (days)	10.18 ± 1.50	10.07 ± 1.64	9.62 ± 1.46	<0.01^de^
Dosage of rFSH (ampules)	29.00 ± 6.79	27.01 ± 4.53	25.92 ± 7.91	<0.01
Serum E2 level (pg/ml)	4,982.93 ± 2,792.94	5,471.74 ± 2,890.84	5,623.62 ± 2,614.19	<0.01^cd^
Serum P level (ng/ml)	1.26 ± 0.75	1.33 ± 0.72	1.37 ± 0.63	NS
No. of oocytes retrieved	14.22 ± 6.95	15.19 ± 7.11	15.53 ± 7.63	<0.01^cd^
No. of available embryos	5.82 ± 3.53	6.10 ± 3.44	6.44 ± 4.16	<0.01
Endometrial thickness at transfer (mm)	11.17 ± 2.40	10.88 ± 2.22	10.95 ± 2.35	NS
Type of transfers, n, (%)				<0.01^cde^
Only a fresh transfer	767 (50.36)	754 (44.96)	146 (38.62)	
Freeze-all and frozen transfer(s)	284 (18.65)	383 (22.84)	95 (25.13)	
One fresh transfer and frozen transfer(s)	472 (30.99)	540 (32.20)	137 (36.24)	
Live-birth rate per clinical pregnancy, n (%)	1,258 (82.6)	1,388 (82.8)	302 (79.9)	NS
OR^a^ (95%Cl)	ref	0.972 (0.801–1.180)	0.910 (0.672–1.232)	
Only a fresh transfer	688 (89.70)	671 (88.99)	126 (86.30)	NS
OR^a^ (95%Cl)	ref	0.883 (0.634–1.230)	0.731 (0.425–1.258)	
Freeze-all and frozen transfer(s)	242 (85.21)	330 (86.16)	81 (85.26)	NS
OR^a^ (95%Cl)	ref	1.045 (0.665–1.642)	1.153 (0.583–2.282)	
One fresh transfer and frozen transfer(s)	328 (69.49)	387 (71.67)	95 (69.34)	NS
OR^a^ (95%Cl)	ref	1.098 (0.831–1.450)	1.047 (0.683–1.607)	

rFSH, recombinant follicle stimulating hormone; E2, estradiol; P, progesterone; NS, not significant; ^a^adjustment with multivariate logistic regression; ^c^E2D <30 pg/ml vs. 30–55 pg/ml; ^d^E2D <30 pg/ml vs. >55 pg/ml; ^e^E2D 30–55 pg/ml vs. >55 pg/ml.

**Figure 2:**
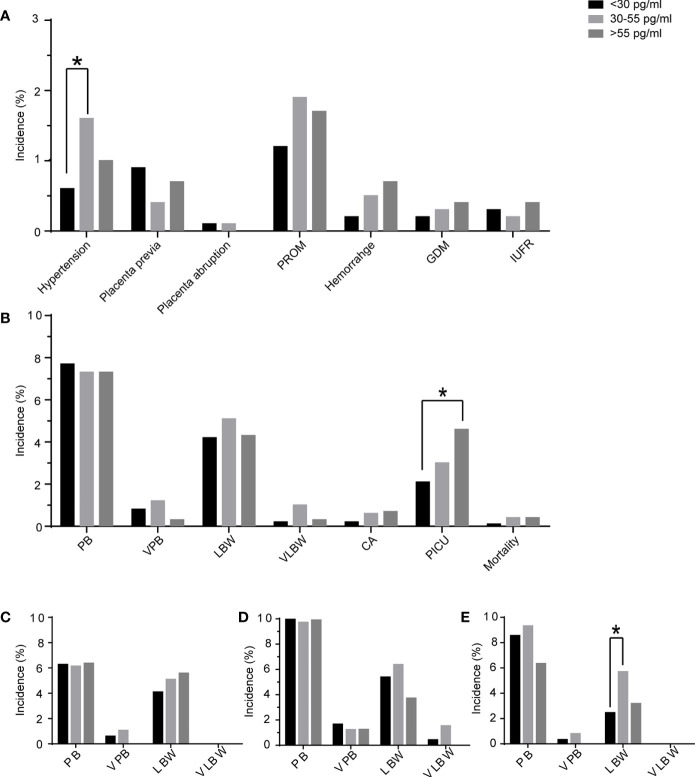
The maternal complications and perinatal outcomes of the patients according to E2D. **(A)** maternal complications of patients with E2D <30, 30–55, >55 pg/ml; **(B)** perinatal outcomes of patients with E2D <30, 30–55, >55 pg/ml; **(C)** perinatal outcomes in the subgroup of only one fresh transfer; **(D)** perinatal outcomes in the subgroup of freeze-all + frozen transfer(s); **(E)** perinatal outcomes in the subgroup of one fresh transfer + frozen transfer(s); PROM, premature rupture of membranes; GDM, gestational diabetes mellitus; IUFR, intrauterine fetal restriction; PB, preterm birth; VPB, very preterm birth; LBW, low birth weight; VLBW, very low birth weight; CA, congenital anomaly; PICU, pediatric intensive care unit; *, significant difference.

The obstetrics and perinatal outcomes are shown in **Table 3** and **Figure 2**. The patients with E2D <30 pg/ml had a lower risk of hypertension disorders compared to those with E2D 30–55 pg/ml (0.56 vs. 1.59%, *P* = 0.04). The risks of placenta previa, placenta abruption, premature rupture of membrane, hemorrhage, gestational diabetes mellitus, and intrauterine fetal restriction were similar among the three E2D groups. No differences were found in the gestational week, percentages of PTB and VPTB, birth weight, percentages of LBW and VLBW, delivery mode, or sex of newborn. The newborns in the group with E2D <30 pg/ml had a lower risk of PICU admission compared to those in the group with E2D >55 pg/ml (2.07 vs. 4.64%, *P* = 0.04). There were no differences in the risks of congenital anomalies or mortality among the three groups.

**Table 3:** The maternal and neonatal outcomes.

	E2 <30 pg/ml	E2D 30–55 pg/ml	E2D >55 pg/ml	*P* value
No. of deliveries	1,258	1,388	302	
Maternal outcomes				
Hypertension, n (%)	7 (0.56)	22 (1.59)	3 (0.99)	0.04^c^
Placenta previa, n (%)	11 (0.87)	6 (0.43)	2 (0.66)	NS
Placenta abruption, n (%)	1 (0.08)	1 (0.07)	–	–
PROM, n (%)	15 (1.19)	26 (1.87)	5 (1.66)	NS
Hemorrhage, n (%)	2 (0.16)	6 (0.43)	2 (0.66)	NS
GDM, n (%)	2 (0.16)	4 (0.29)	1 (0.33)	NS
IUFR, n (%)	4 (0.32)	3 (0.22)	1 (0.33)	NS
Neonatal outcomes				
Gestational week (week)	38.61 ± 1.54	38.52 ± 1.64	38.53 ± 1.31	NS
MD^b^ (95%Cl)	ref	-0.072 (-0.222~0.078)	-0.052 (-0.306~0.202)	
Preterm birth, n (%)	96 (7.63)	109 (7.85)	22 (7.28)	NS
OR^a^ (95%Cl)	Ref	0.972 (0.726–1.301)	0.869 (0.531–1.421)	
Very preterm birth, n (%)	9 (0.72)	14 (1.01)	1 (0.33)	NS
Adjustment NA	–	–	–	
Birth weight (g)	3,312.13 ± 478.21	3,290.51 ± 534.13	3,344.65 ± 477.39	NS
MD^b^ (95%Cl)		-22.69 (-70.60~25.23)	39.42	
	ref		(-41.72~120.57)	
Low birth weight, n (%)	49 (3.90)	77 (5.55)	13 (4.30)	NS
OR^a^ (95%Cl)	ref	1.344 (0.928–1.947)	1.089 (0.578–2.052)	
Very low birth weight, n (%)	2 (0.16)	12 (0.86)	1 (0.33)	NS
Adjustment NA	–	–	–	
Delivery mode, n (%)				NS
Cesarean section	1,174 (93.32)	1,294 (93.23)	281 (93.05)	
OR^a^ (95%Cl)	ref	0.981 (0.712–1.352)	0.963 (0.562–1.648)	
Vaginal delivery	84 (6.68)	94 (6.77)	21 (6.95)	
OR^a^ (95%Cl)	ref	1.019 (0.740–1.404)	1.039 (0.607–1.778)	
Sex of newborn, n (%)				NS
Male	680 (54.05)	759 (54.68)	163 (53.97)	
OR^a^ (95%Cl)	ref	1.013 (0.862–1.189)	1.000 (0.767–1.305)	
Female	578 (45.95)	629 (45.32)	139 (46.03)	
OR^a^ (95%Cl)	ref	0.987 (0.841–1.160)	1.000 (0.767–1.305)	
Congenital anomaly, n (%)	3 (0.24)	8 (0.58)	2 (0.66)	NS
PICU attempt, n (%)	26 (2.07)	42 (3.03)	14 (4.64)	0.04^d^
Mortality, n (%)	1 (0.08)	5 (0.36)	1 (0.33)	NS

PROM, premature rupture of membranes; GDM, gestational diabetes mellitus; IUFR, intrauterine fetal restriction; PICU, pediatric intensive care unit; NS, not significant; NA, not available; MD, mean difference; ^a^adjustment with multivariate logistic regression; ^b^adjustment with multivariate general linear model; ^c^E2D <30 pg/ml vs. 30–55 pg/ml; ^d^E2D <30 pg/ml vs. >55 pg/ml.

Patients were sub-divided into three subgroups according to the mode of transfer. The results of subgroup analyses are shown in **Tables 4**–**6**. In the subgroup of patients who underwent one fresh transfer only, and in the subgroup of patients who underwent the strategy of freeze-all + subsequent frozen transfer(s), E2D had no significant effect on the gestational week, percentages of PTB and VPTB, birth weight, percentages of LBW and VLBW, delivery mode, or sex of newborn. However, in the subgroup of patients who underwent one fresh transfer + subsequent frozen transfer(s), E2D 30–55 pg/ml was associated with a lower gestational week compared to E2D <30 pg/ml (38.35 ± 1.58 vs. 38.64 ± 1.43, *P* = 0.0497). E2D 30–55 pg/ml was also associated with a higher risk of LBW compared to E2D <30 pg/ml (5.68 vs. 2.44%, *P* = 0.01).

**Table 4:** The maternal and neonatal outcomes in patients with only fresh transfers.

	E2 <30 pg/ml	E2D 30–55 pg/ml	E2D >55 pg/ml	*P* value
No. of clinical pregnancies	767	754	146	
No. of deliveries	688	671	126	
Gestational week (week)	38.64 ± 1.53	38.67 ± 1.56	38.65 ± 1.23	NS
MD^b^ (95%Cl)	ref	0.032 (-0.177~0.240)	0.015 (-0.363~0.392)	
Preterm birth, n (%)	43 (6.25)	41 (6.11)	8 (6.35)	NS
OR^a^ (95%Cl)	ref	0.910 (0.573-1.445)	1.037 (0.469–2.294)	
Very preterm birth, n (%)	4 (0.58)	7 (1.04)	–	NS
Adjustment NA	–	–	–	
Birth weight (g)	3,259.00 ± 455.40	3,244.51 ± 499.34	3,265.61 ± 489.67	NS
MD^b^ (95%Cl)	ref	-23.89 (-88.09~40.32)	4.16(-112.20~120.53)	
Low birth weight, n (%)	28 (4.07)	34 (5.07)	7 (5.56)	NS
OR^a^ (95%Cl)	ref	1.204 (0.707–2.051)	1.422 (0.597–3.386)	
Very low birth weight, n (%)	1 (0.15)	7 (1.04)	1 (0.79)	NS
Adjustment NA	–	–	–	
Delivery mode, n (%)				NS
Cesarean section	634 (92.15)	615 (91.65)	122 (96.83)	
OR^a^ (95%Cl)	ref	0.944 (0.624–1.427)	2.378 (0.836–6.763)	
Vaginal delivery	54 (7.85)	56 (8.35)	4 (3.17)	
OR^a^ (95%Cl)	ref	1.059 (0.701–1.601)	0.420 (0.148–1.196)	
Sex of newborn, n (%)				NS
Male	355 (51.60)	345 (51.42)	65 (51.59)	
OR^a^ (95%Cl)	ref	1.020 (0.814–1.280)	1.010 (0.673–1.514)	
Female	333 (48.40)	326 (48.58)	61 (48.41)	
OR^a^ (95%Cl)	ref	0.980 (0.782–1.229)	0.991 (0.660–1.486)	

NS, not significant; NA, not available; MD, mean difference; ^a^adjustment with multivariate logistic regression; ^b^adjustment with multivariate general linear model.

**Table 5:** The maternal and neonatal outcomes in patients with freeze-all and subsequent frozen transfer(s).

	E2 <30 pg/ml	E2D 30–55 pg/ml	E2D >55 pg/ml	*P* value
No. of clinical pregnancies	284	383	95	
No. of deliveries	242	330	81	
Type of embryo transferred, n (%)				NS
Day 3 cleavage-stage	89 (36.78)	111 (33.64)	34 (41.98)	
Day 5 blastocyst	153 (63.22)	219 (66.36)	47 (58.02)	
Gestational week (week) MD^b^ (95%Cl)	38.50 ± 1.73 ref	38.42 ± 1.85 -0.073 (-0.442~0.297)	38.38 ± 1.62 -0.111 (-0.671~0.449)	NS
Preterm birth, n (%) OR^a^ (95%Cl)	25 (10.33) ref	32 (9.70) 0.950 (0.542–1.664)	8 (9.88) 0.809 (0.338–1.934)	NS
Very preterm birth, n (%) Adjustment NA	4 (1.65) -	4 (1.21) -	1 (1.23) -	NS
Birth weight (g) MD^b^ (95%Cl)	3,346.28 ± 534.53 ref	3,336.16 ± 575.51 -8.95 (-122.1~104.2)	3,407.33 ± 458.09 62.22 (-109.0~233.5)	NS
Low birth weight, n (%) OR^a^ (95%Cl)	13 (5.37) ref	21 (6.36) 1.080 (0.524–2.227)	3 (3.70) 0.736 (0.200–2.704)	NS
Very low birth weight, n (%) Adjustment NA	1 (0.41) -	5 (1.52) -	- -	NS
Delivery mode, n (%)				NS
Vaginal delivery	13 (5.37)	20 (6.06)	17 (11.11)	
OR^a^ (95%Cl)	ref	0.999 (0.468–2.132)	2.527 (0.961–6.646)	
Cesarean section OR^a^ (95%Cl)	229 (94.63) ref	310 (93.94) 1.001 (0.469–2.135)	72 (88.89) 0.396 (0.150–1.041)	
Sex of newborn, n (%)				NS
Male	137 (56.61)	189 (57.27)	54 (66.67)	
OR^a^ (95%Cl)	ref	0.990 (0.697–1.406)	1.577 (0.901–1.406)	
Female	105 (43.39)	141 (42.73)	27 (33.33)	
OR^a^ (95%Cl)	ref	1.010 (0.711–1.435)	0.634 (0.362–1.110)	

NS, not significant; NA, not available; MD, mean difference; ^a^adjustment with multivariate logistic regression; ^b^adjustment with multivariate general linear model.

**Table 6:** The maternal and neonatal outcomes in patients with one fresh transfer and frozen transfer(s).

	E2 <30 pg/ml	E2D 30–55 pg/ml	E2D >55 pg/ml	*P* value
No. of clinical pregnancies	472	540	137	
No. of deliveries	328	387	95	
Type of embryo transferred, n (%)				0.03^d^
Day 3 cleavage-stage Day 5 blastocyst	119 (36.28) 209 (63.72)	115 (29.72) 272 (70.28)	40 (42.11) 55 (57.89)	
Gestational week (week) MD^b^ (95%Cl)	38.64 ± 1.43 ref	38.35 ± 1.58 -0.285 (-0.556~-0.014)	38.52 ± 1.11 -0.102 (-0.529~0.325)	0.0447^c^ 0.0497^c^
Preterm birth, n (%) OR^a^ (95%Cl)	28 (8.54) ref	36 (9.30) 1.079 (0.636–1.828)	6 (6.32) 0.741 (0.293–1.878)	NS
Very preterm birth, n (%) Adjustment NA	1 (0.30) -	3 (0.78) -	- -	NS
Birth weight (g) MD^b^ (95%Cl)	3,398.53 ± 468.19 ref	3,333.48 ± 551.62 -66.99 (-160.45~26.47)	3,394.77 ± 467.79 -0.84 (-148.06~146.39)	NS
Low birth weight, n (%) OR^a^ (95%Cl)	8 (2.44) ref	22 (5.68) 2.436 (1.060–5.600)	3 (3.16) 1.430 (0.367–5.576)	NS 0.01^c^
Very low birth weight, n (%)	–	–	–	NA
Adjustment NA	–	–	–	
Delivery mode, n (%)				NS
Vaginal delivery OR^a^(95%Cl)	17 (5.18) ref	18 (4.65) 0.970 (0.473–1.992)	8 (8.42) 1.572 (0.583–4.240)	
Cesarean section OR^a^ (95%Cl)	311 (94.82) ref	369 (95.35) 1.031 (0.502–2.116)	87 (91.58) 0.636 (0.236–1.715)	
Sex of newborn, n (%)				NS
Male OR^a^ (95%Cl)	188 (57.32) ref	225 (58.14) 0.999 (0.730–1.368)	44 (46.32) 0.666 (0.411–1.078)	
Female OR^a^ (95%Cl)	140 (42.68) ref	162 (41.86) 1.001 (0.731–1.370)	51 (53.68) 1.502 (0.928–2.432)	

NS, not significant; NA, not available; MD, mean difference; ^a^adjustment with multivariate logistic regression; ^b^adjustment with multivariate general linear model; ^c^E2D <30pg/ml vs. 30–55 pg/ml; ^d^E2D 30–55 pg/ml and >55pg/ml.

## Discussion

This study compared the ovarian stimulation performance, live-birth rate, and maternal and perinatal outcomes of patients with E2D <30, 30–55, and >55 pg/ml. We found that E2D was associated with ovarian response, but did not influence the live-birth rate. The patients with E2D <30 pg/ml were less likely to have hypertension disorders and their babies had a lower risk of PICU admission. Subgroup analysis showed that, in patients with one fresh transfer + subsequent frozen transfer(s), E2D 30–55 pg/ml was associated with a higher risk of LBW.

Down-regulation is a common practice in IVF treatment. Previous randomized trials have demonstrated that down-regulation with GnRH-a reduces the cycle cancellation rate and increases the number of available oocytes/embryos (2, 18). E2D is an important parameter for evaluating whether down-regulation has been achieved; however, there is still no consensus on the E2D cut-off point. Many studies have not reported their definitions of down-regulation. Antoine et al. defined it as a serum E2D <30 pg/ml (18). Barash et al. used an E2D of 55 pg/ml as the cut-off point, and down-regulation was achieved in 77% of the cycles in their study (19). Moreover, the degree of down-regulation has been less well studied. Research has shown that the degree of pituitary suppression had no significant effects on pregnancy or live birth (1, 20). However, these studies are relatively old and included only cycles with fresh transfers, which do not completely represent the quality of all oocytes obtained in a stimulated cycle. Our recent study found that patients with E2D <30 or 30-55 pg/ml had higher cumulative live birth rates per stimulated cycle, compared to patients with E2D >55 pg/ml (3). This result indicates that down-regulation with E2D <30 or 30–55 pg/ml improves folliculogenesis and subsequent IVF outcomes. Furthermore, to the best of our knowledge, there is a severe lack of clinical evidence on maternal and perinatal outcomes associated with different down-regulation conditions.

This study showed that the degree of down-regulation was negatively associated with ovarian response. With the decrease of E2D, more exogenous gonadotropin was needed, but the peak E2 level was lower. A lower E2D was also associated with a longer duration of stimulation and decreased numbers of oocytes and embryos; however, these differences were slight and of low clinical significance. The underlying mechanism is that the pituitary gland needs more time and more aggressive stimulation to recover from a profoundly suppressed condition, before the initiation of folliculogenesis.

Our previous study demonstrated that sufficient down-regulation (E2D <55 pg/ml) increases the implantation potential, in terms of cumulative clinical pregnancy rate per retrieval cycle (3). The present study further evaluated the effect of E2D on the maintenance potential of pregnancy. The patients with different E2D values had comparable live-birth rates. The clinical significance of this result is that it can help clinicians in daily counselling and in making prognoses. Insufficient down-regulation (E2D >55 pg/ml) indicates a lower chance of pregnancy, but the risk of pregnancy loss does not increase if a pregnancy has been achieved.

Since the first IVF baby (21), the number of children born after IVF has risen rapidly over the past 40 years. Globally, increasing attention is being paid to the safety of IVF, in terms of obstetrics and perinatal outcomes. However, the results of previous studies are conflicting. Some studies have reported that IVF is a generally safe procedure (22–24). In contrast, some studies found increased risks for newborns and/or mothers after IVF (9, 25). Superovulation or culture conditions may have contributed to these adverse outcomes (26). Regarding maternal complications, we found that E2D <30 pg/ml was associated with a lower risk of hypertension disorders of pregnancy. The quality of oocytes/embryos may contribute to the pathogenesis of hypertension disorders. Indeed, abnormal cytotrophoblasts can cause inadequate spiral artery remodeling and atherosis, resulting in ischemia and hypoxia of the placenta, which is central to the pathogenesis of this disease (27). Regarding perinatal outcomes, we found that the babies of patients with E2D <30 pg/ml were less likely to require hospitalization in the PICU. A possible rationale is that a profound down-regulation may improve folliculogenesis and the subsequent embryo/fetus viability, thus reducing the risk of PICU admission.

E2D 30–55 pg/ml was found to have adverse effects on neonatal outcomes in the subgroup of patients who underwent one fresh transfer + subsequent frozen transfer(s). The mean gestational week of patients with E2D 30–55 pg/ml was 0.29 weeks (about 2 days) earlier than that of patients with E2D <30 pg/ml. This difference was slight and of low clinical significance. E2D 30–55 pg/ml resulted in a 3.24% increase in the incidence of LBW compared to E2D <30 pg/ml. This result suggests that E2D affects oocyte/embryo quality, which may affect the birth weight. Based on the transfer strategy at our center, best-quality embryos were used first in fresh cycles. Thus, in the subgroup of patients who underwent one fresh transfer + subsequent frozen transfer(s), best-quality embryos were initially transferred, but they did not result in pregnancies. This indicates that the implantation potential of embryos from these patients was relatively low. In addition, the embryos transferred in the subsequent frozen cycle(s) after the initial fresh cycle were generally of poorer quality than those used in the fresh cycle. Therefore, in patients whose embryos are of relatively low quality, E2D 30–55 pg/ml may be associated with LBW.

A limitation of this study is its retrospective nature. Confounders may affect the results, though multivariate models were used to adjust for several confounders. Although this study included a large number of patients, the incidences of several maternal and perinatal diseases were very low, which may have led to less power to detect differences between the groups. A further prospective study with sufficient numbers of patients should be performed to confirm our findings. A strength of this study is its large sample size. In addition, this study analyzed only singleton pregnancies, which can allow the effect of twin-associated complications on the outcomes to be ruled out.

In conclusion, this study showed that down-regulation had no effect on the live-birth rate per clinical pregnancy. Patients with E2D <30 pg/ml may have advantages regarding lower risks of both maternal hypertension and newborns PICU admission. E2D 30–55 pg/ml may be associated with LBW in patients with relatively low quality embryos.

## Data Availability Statement

The original contributions presented in the study are included in the article/**Supplementary Material**. Further inquiries can be directed to the corresponding author.

## Ethics Statement

The studies involving human participants were reviewed and approved by the Institution Review Board (IRB) of Tongji Hospital. The patients/participants provided their written informed consent to participate in this study.

## Author Contributions

All authors contributed to the study conception and design. Data collection and analysis were performed by LJ, JA, YZ, and BC. Data interpretation was performed by LJ, JA, LW, and XD. The first draft of the manuscript was written by LJ and JA, and all authors commented on previous versions of the manuscript. All authors agreed to be accountable for the content of the work. All authors contributed to the article and approved the submitted version.

## Conflict of Interest

The authors declare that the research was conducted in the absence of any commercial or financial relationships that could be construed as a potential conflict of interest.

## Supplementary Material

Supplementary Figure 1 The restricted cubic spline analysis of the association between E2D and live birth.
