# Supplementary figures and images for: The Impact of Down-Regulation on Obstetrics and Perinatal Outcomes in Singleton Pregnancies After In Vitro Fertilization
Source: Front Endocrinol (Lausanne). 2021 Mar 10;12:622081. doi: 10.3389/fendo.2021.622081 (PMC7988215; doi:10.3389/fendo.2021.622081)

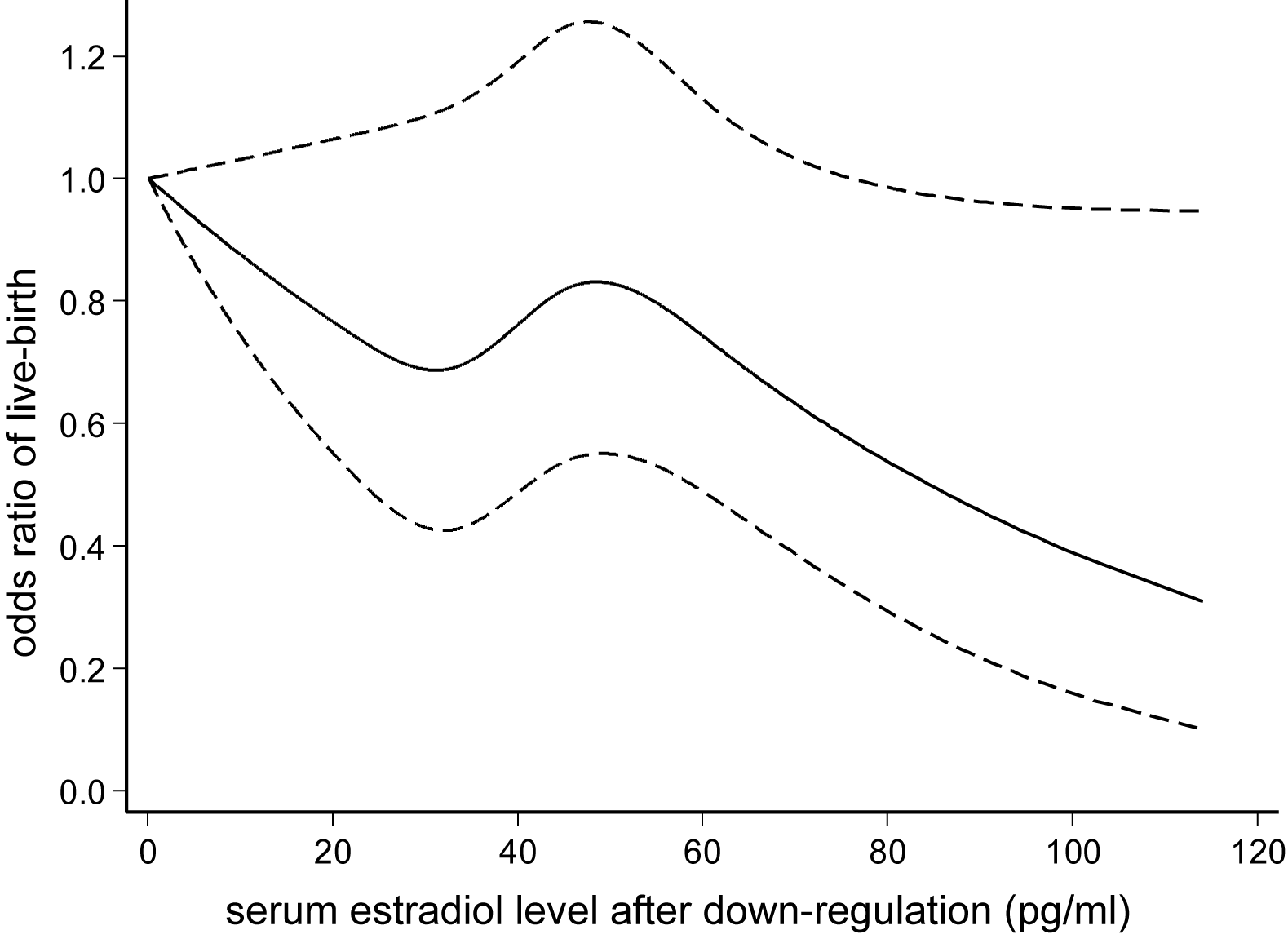

Supplement: Supplementary Figure 1 — The restricted cubic spline analysis of the association between E2D and live birth. [file Image_1.tif]
